# Landscape of WASH-relevant Training for Humanitarian Emergencies

**DOI:** 10.1371/currents.dis.a0351bdc63f477245858caa7638e8525

**Published:** 2015-05-11

**Authors:** Caetano Dorea

## Abstract

Background: Both employed humanitarian personnel as well as those seeking to start a career as an aid worker are often provided with or seek training on the theme of humanitarian water, sanitation, and hygiene (WASH). The objective of this study was to conduct a landscaping exercise of the available WASH-relevant training for humanitarian emergencies.

Methods: An open internet search was performed with specific terms related to humanitarian WASH. Retained search results included those training opportunities (including past ones) that were themed around or with a mentioned relevance to humanitarian WASH.

Results and Discussion: A total of 42 training courses relevant to humanitarian emergency WASH were retained. In addition to the more generic/introductory trainings, some provided thematic variations such as coordination of WASH responses, project management, risk reduction, information, education and communication (IEC), and complex emergencies. Timely topics such as urban WASH, Ebola, and WASH innovations were also observed indicating the responsiveness of the training providers to the changing needs of humanitarian WASH response programmes. This survey also revealed a large variety in terms of target audience, duration, fees, location, and language of courses. There was no centralised listing of courses available on the Internet. Limitations of this exercise were also discussed.

## Introduction

Humanitarian emergencies, whether natural or manmade, often require relief interventions in water, sanitation, and hygiene (WASH) to prevent the spread of diarrhoeal diseases. Such illnesses can be one of the major contributors to the overall morbidity and mortality rates following a disaster^1^^,^ 2. Typically, when the (re-)establishment of adequate WASH infrastructures is beyond the capacity of local authorities, aid from external relief agencies is warranted. In addition to dedicated equipment and other relief items, such organisations use specialised relief personnel to coordinate and deliver WASH interventions during recovery efforts. It is often the case that such personnel are either expatriate relief workers or locally-hired staff. In both cases, humanitarian WASH training is many times offered or required. The anecdotally romantic view that a relevant expertise and lots of goodwill were sufficient to secure a posting as an aid worker no longer holds true. Qualified personnel (e.g. engineers and health professionals) interested in becoming aid workers now seek such training to further specialise their skills and learn more about humanitarian work.

In 2007 Oxfam International^3^ reported that over the previous two decades the total number of natural disasters has increased four-fold and that the number of affected people from such events has risen on average from 174 million to 250 million a year. Moreover, since the end of the Cold War in 1991, an increase in the number of violent manmade conflicts has also been noticed^4^ affecting a growing number of civilian populations. Evidence of this changing landscape may also be found in the overall increasing trend humanitarian funding and response personnel with an estimated annual growth rate of 6 percent^5^, as pointed out by Walker et al.^6^. The increase in such statistics will likely reflect an increase of humanitarian support WASH personnel, given the importance (and near ubiquity) of WASH interventions during such catastrophic events. Adequate training programmes are thus warranted to ensure professional development, accountability to beneficiaries, and building trust with donors.

Walker and Russ^7^ conducted a scoping study on the professionalisation of the humanitarian sector and presented a database of mostly non-specific humanitarian training that was available. More recently, Jacquet et al.^8^ presented a mainly literature-based survey (albeit non-systematically), but also with a broad “humanitarian response” remit and did not identify WASH-specific training. Furthermore, it concentrated on more traditional (“in-class”) type training, as distance learning options (i.e. online or paper-based) were not included in their assessment. The objective of this study was to conduct a landscaping exercise of the available WASH-relevant training for humanitarian emergencies.

## Methods

An open internet search was performed in English with Google web search engine (www.google.ca) using a combination of the terms “water,” “sanitation,” “hygiene,” “WASH,” “watsan,” “humanitarian,” “emergencies,” “disasters,” “relief,” “course,” and “training” with no search operators. Due to the large number of results obtained through open internet searches (sometimes over 40,000,000), only the first 50 results for each search were evaluated for inclusion in the study. In addition to the primary search results, secondary references were also followed up. These searches were conducted between December 2014 and February 2015. Websites were typically visited only once for information retrieval. Retained search results included those training opportunities (including past ones) that were themed around water, sanitation and/or hygiene dedicated to or with mentioned relevance to humanitarian relief. Generic (i.e. non-specific to emergencies) WASH trainings were not included in this exercise. When possible the name, organiser, location, delivery mode (e.g. presential or online), duration, fees (converted to USD), and language (assumed to be the same of the information source when not explicit) were noted.

## Results

A total of 42 training courses relevant to humanitarian emergency WASH were retained for this exercise. Whereas this search was not exhaustive with regards to the existing plethora of (generic) WASH training courses, it identified emergency WASH-related opportunities that are largely advertised to the general public. Although some courses were also part of a larger academic postgraduate study programme. Many organisations can offer “in-house” trainings (sometimes contracted out to third parties) that are not advertised and are restricted to their employees. Conferences and workshops with relevance to humanitarian emergency WASH also can provide valuable learning opportunities, which were not covered by this study. The open internet search also revealed “general” WASH-relevant courses that were not specific to humanitarian emergencies. Whilst there can be a strong overlap of content that is applicable to humanitarian contexts, such results were not within the scope of the performed search. The retained search results were categorised according to their delivery mode. For presential courses, the reported durations varied from 1 day to 10 months. Online trainings were anywhere between 2 hours of duration to up to 150 contact hours. Their characteristics were summarised in Tables 1 and 2.

**Table 1. Summary of presential (on-site) humanitarian WASH-relevant training courses sorted by duration in days (unless otherwise stated).** NS = not stated.

Name	Organiser	Location	Duration	Fee (USD)	Language
Introduction to Hygiene Promotion in Emergencies	RedR-UK	London, UK	1	155 to 379	English
Sustainable Sanitation in Emergencies and Reconstruction Situations	German Toilet Organization	NS	2	NS	German
Hygiene Promotion and Behaviour Change	German Toilet Organization	Berlin, Germany	2	NS	German
Emergency Water, Sanitation and Hygiene (WASH) Engineering: Hands-on Weekend	Engineers Without Borders - UK	Wakefield, UK	3	147	English
Public Health Promotion in Emergencies	RedR-India	NS	3	NS	Hindi
Hygiene Promotion in Emergencies	RedR-India	Guwahati, India	4	343 to 526	English
WASH Essentials in Emergencies	RedR-UK	Nairobi, Kenya	5	246	English
Hygiene Promotion Training for Trainers for Emergency Contexts	IDEAL Public Health and Development Consultancy	Nairobi, Kenya	5	600	English
Master Essential Rural and Periurban WASH Techniques	Bioforce	Bamako, Mali	5	683	French
Coordinating Hygiene Promotion & Favour Community Participation	Bioforce	Bamako, Mali	5	683	French
Managing Emergency Sanitation & Sustainable Sanitation	Bioforce	Bamako, Mali	5	683	French
Technical Training in Water & Sanitation	Bushproof	Antananarivo, Madagascar	5	1708	English
Urban WASH in Emergencies	RedR-UK	London, UK	5	860 to 2080	English
WASH in Emergencies	RedR-UK	Khartoum, Sudan	5	860 to 2080	English
Advanced Technologies for Water and Sanitation	Cranfield University	Cranfield, UK	5	1966 to 2151	English
Emergency Water Supply and Environmental Sanitation	Cranfield University	Cranfield, UK	5	1966 to 5151	English
Water, Sanitation, and Hygiene (WASH) In Emergencies Training	Department of Health (Gov. of the Rep. of the Philippines)	NS	5	NS	English
WASH in Emergency Training	Roberto Saltori (Private Consultant)	Sando, Sweden	5	NS	English
WatSan in Emergency Training	Austrian Red Cross	Laubegg, Austria	7	Free	English
Environmental Health in Emergencies	RedR-India	Pune, India	7	343 to 565	English
Engineering in Emergencies	RedR-India	Kathmandu, Nepal	7	424 to 750	English
NTS 004 WASH in emergencies: Risk Reduction	Netwas International	Nairobi, Kenya	7	949 to 1680	English
Water, Sanitation & Hygiene in Emergencies	RedR-Australia	Dookie, Australia	7	1234 to 1912	English
Water and Sanitation Engineering from Emergency Towards Development	University of Neuchâtel & the International Committee of the Red Cross	Neuchâtel, Switzerland	7	2151	English
Coordinating Emergency WASH Responses: Lessons Learnt from Western and Central Africa	Bioforce	Bamako, Mali	10	1708	French
Health Emergencies in Large Populations (HELP)	Johns Hopkins University & International Committee of the Red Cross	Baltimore, USA	10	1899 to 4930	English
Specialisation in Ebola Management for Log/WASH Professionals	Bioforce	Lyon, France	13	569	English
Learn the Specific Competencies of WASH Professionals	Bioforce	Lyon, France & Bamako, Mali	20 to 27	2074	French
Water Supply and Sanitation in Emergencies	University of Copenhagen	Copenhagen, Denmark	4 weeks	2829 to 3824	English
Advanced Master’s Degree in Humanitarian WASH	International Institute for Water and Environmental Engineering	Ouagadougou, Burkina Faso	10 months	6688 to 7895	English
Water, Sanitation and Hygiene	RedR-Malaysia	NS	NS	NS	English
Water and Ecological Sanitation in Crisis Contexts	Groupe u.r.d.	NS	NS	NS	French

**Table 2. Summary of online humanitarian WASH-relevant training courses sorted by duration in hours.** NS = not stated. * indicates duration expressed as the reported "contact hours" (i.e. including self study hours).

Name	Organiser	Duration	Fee (USD)	Language
Development, Disasters and Sanitation	Loughborough University	2	15	English
Information, Education and Communication (IEC) in WASH emergencies	Oxfam	6	Free	English
Technical Project Management (TPM) in WASH Emergencies	Oxfam	14	Free	English
Emergency Sanitation (CVP282)	Loughborough University	150*	1693	English
Emergency Water Supply (CVP281)	Loughborough University	150*	1693	English
Emergency Water Supply and Sanitation: A WELL Course	Loughborough University	NS	Free	English
Water Sanitation Needs in Complex Humanitarian Emergencies	Johns Hopkins University	NS	Free	English
Refugee Health Care	Johns Hopkins University & International Committee of the Red Cross	NS	Free	English
Public Health e-learning	UNCHR	NS	Free	English
Health in Humanitarian Crises	Advanced Training Program on Humanitarian Action (ATHA)	NS	Free	English

## Discussion

The content of most of the available trainings was centered on notions of emergency water supply (e.g. water quality, treatment, distribution, etc.), sanitation (e.g. latrine types, faecal sludge management, etc.), and hygiene (e.g. handwashing, hygiene promotion methods and approaches, behaviour change, etc.), as well as overarching themes (e.g. SPHERE Standards, diarrhoeal disease transmission, etc.). However, some courses provided thematic variations such as coordination of WASH responses, project management, risk reduction, information, education and communication (IEC), and complex emergencies. Timely topics such as urban WASH, Ebola, menstrual hygiene management, and WASH innovations were also observed indicating the responsiveness of the training providers to the changing needs of humanitarian WASH response programmes. There did not seem to be any obvious topic omission. However, there could be specialised themes that were not covered in the training opportunities that were largely aimed at a rather generic humanitarian audience.

Fees for the training courses also varied substantially. Whereas some online ones were offered free of charge, others cost between 15 and 7895 USD. Variation between courses are thought to be related to the duration and venue of the trainings. Price variations were also observed within courses according to several factors such as inclusion of accommodation and meals, student status, group registration, professional status (e.g. NGO, local government, etc.), academic credit or non-credit bearing, residency status, medical insurance, etc.

This scoping exercise revealed mainly training opportunities in English, as it was biased by the Language in which the open internet search that was performed. It is possible that a search in other languages would reveal an even greater number and variety. Although all of the information retrieved for this exercise was in English, some course information stated content delivery in languages other than English (e.g. French, German, and Hindi). Moreover, given that some organisations offered bespoke on demand courses, it is likely that these can be offered in other languages (or dialects).

Although the training courses were largely advertised to the general public, their intended audience varied. While the target clientele was not consistently indicated, the training opportunities were aimed at humanitarian WASH professionals, engineers, technicians, public health/medical professionals, logisticians, project managers, programme coordinators, and graduate students. Previous experience was generally not a requirement. Some courses required a minimum number of registrations in order to take place.

In some instances, distribution certificates of completion were explicitly mentioned. Nonetheless, this is thought to be the norm with such type of trainings. No trainings were part of a recognised humanitarian WASH certification, as in general humanitarian occupational standards do not exist^7^. In this study the terms “certificates” and “certification” are used as defined elsewhere^7^. The professionalisation of the humanitarian sector is currently being discussed^6^. Relevant professional qualifications for WASH professionals are varied (e.g. engineers, social sciences, environmental or public health related disciplines), thus the professionalisation of humanitarian WASH workers may be a challenge. Moreover, many of such professionals are frequently certified through other professional organisations usually at a national level. On the other hand, minimum standards in humanitarian WASH interventions do exist^9^ and these are largely reflected on the delivered course content that was available. The information on course curricula that was analysed was limited to that available on searched websites. Therefore, it was not possible to identify and distil common points with regards to core competencies being developed and how they are being delivered. However, given the broad spectrum of relief activities that are included under the WASH acronym, it may be challenging to find a consensus with regards to core competencies in this theme.

This landscaping exercise revealed a large variety (in many aspects) of courses themed around humanitarian emergency WASH. There was no centralised listing of courses available on the Internet for those seeking training on the subject. Popular humanitarian-focused information portals such as ReliefWeb (www.reliefweb.int) that already host training listings in addition to other information (e.g. situation updates, country reports, job postings, etc.) could be potential candidates for a centralised repository. To this end, this survey can also be useful to those seeking WASH training to either gain awareness on the topic or to refresh their knowledge, as well as a starting point for those seeking to initiate a career in this field.

## Appendix 1

Websites visited during this study for training course information retrieval: Download PDF
